# Dengue 2 infection of HepG2 liver cells results in endoplasmic reticulum stress and induction of multiple pathways of cell death

**DOI:** 10.1186/1756-0500-6-372

**Published:** 2013-09-14

**Authors:** Chutima Thepparit, Atefeh Khakpoor, Sarawut Khongwichit, Nitwara Wikan, Chanida Fongsaran, Pimjai Chingsuwanrote, Patcharee Panraksa, Duncan R Smith

**Affiliations:** 1 Molecular Pathology Laboratory, Institute of Molecular Biosciences, Mahidol University, 25/25 Phuttamontol Sai 4, Salaya, Nakorn Pathom 73170, Thailand; 2 Center for Emerging and Neglected Infectious Diseases, Mahidol University, Bangkok, Thailand

**Keywords:** Apoptosis, Autophagy, Caspase, Dengue, ER stress liver

## Abstract

**Background:**

A number of studies have implicated the direct involvement of the liver in dengue virus (DENV) infection, and it has been widely shown that liver cells subsequently undergo apoptosis. The mechanism by which liver cells undergo apoptosis in response to DENV infection remains unclear. To provide further information on the mechanism of apoptosis in DENV infected liver cells, HepG2 cells were infected with DENV 2 and analyzed for the induction of ER stress, apoptosis and autophagy.

**Results:**

In response to DENV infection, HepG2 cells showed the induction of both the ER resident unfolded protein response as well as the Noxa/PUMA stress response pathways. Proteolytic activation of caspases 4, 7, 8 and 9 was observed as well as changes in mitochondrial transmembrane potential. Increased monodansylcadaverine staining was observed in DENV infected cells, consistent with the previously reported induction of autophagy.

**Conclusions:**

These results are consistent with a model in which the induction of multiple ER stress pathways is coupled with the induction of multiple cell death pathways as a mechanism to ensure the removal of infected liver cells from the system.

## Background

Dengue virus (DENV; family *Flaviviridae*, genus *Flavivirus*) is the causative agent of dengue, a mosquito-transmitted viral disease. In humans infection results in a wide range of clinical manifestations from a relatively self-limiting febrile illness termed dengue fever (DF) to more severe forms that can threaten the patient’s life through plasma leakage in dengue haemorrhagic fever (DHF) and dengue shock syndrome (DSS) [1]. An estimated 390 million dengue infections are believed to occur each year, of which some nearly 100 million show manifestation of the disease at some severity level [2]. The involvement of the liver in the pathogenesis of dengue is suggested by evidence of hepatomegaly in dengue patients [3] as well as the elevated levels of serum alanine aminotransferase and alkaline phosphatase [4,5]. Direct evidence of the involvement of the liver in the disease arises from studies that have shown the presence of dengue viral antigens in samples of human liver from fatal cases of dengue disease [6-9].

The presence of councilman bodies (believed to be the remains of cells undergoing apoptosis) have also been observed in specimens of liver collected at autopsy [9-11], and both primary kupffer cells and hepatocytes undergo apoptosis in response to DENV infection [12,13], although only the latter cell type is productively infected. Several studies have investigated the mechanism of apoptosis induction in liver cell lines in response to DENV infection, although little consensus has emerged as to which cell death pathway or pathways are triggered, or how the process is initiated [14-18].

We have recently shown in monocytic cells that DENV infection results in the induction of the unfolded protein response (UPR) and the Noxa/PUMA (p53 upregulated modulator of apoptosis) endoplasmic reticulum (ER) stress pathways, and that coupled with this is the activation of both intrinsic and extrinsic apoptosis pathways [19]. Intrinsic apoptosis pathways are primarily mediated through mitochondria and are typified by the proteolytic activation of caspase 9, while extrinsic apoptosis is characterized by the involvement of death receptors and typified by the proteolytic activation of caspase 8 [20].

The primary function of the UPR is to adapt to changes in the environment and reestablish normal ER function [21]. The central mediator of the ER stress response is GRP78 (Glucose regulated protein 78 or BiP, Immunoglobulin heavy chain binding protein) and under normal conditions GRP78 binds to three critical ER transmembrane signaling proteins IRE1 (Inositol-requiring protein 1), ATF6 (Activating transcription factor 6), and PERK (Protein kinase RNA-like endoplasmic reticulum kinase) [22]. These three genes play a critical role in the adaptation mechanism by inducing the transcription of ER resident chaperones, blocking the translation of mRNAs in order to reduce the flux of the newly synthesized proteins to the ER, and increasing the amount of protein degradation [21-23]. Upon ER stress, GRP78 releases IRE1 and PERK leading to homodimerization and autophosphorylation and subsequent activation of each protein. Activated IRE1 excises a 26-nucleotide intron from the XBP-1 (Xbox binding protein) transcript producing a transcription factor that induces the expression of ER resident chaperons or proteins involved in the degradation process [24]. Oligomerized and phosphorylated PERK blocks the translation of most cytoplasmic mRNAs by phosphorylating eukaryotic initiation factor 2α (eIF-2α) and activating the expression of further downstream genes which are primarily involved in the regulation of apoptosis [25]. In contrast, upon release from GRP78, ATF6 is cleaved in the Golgi compartment by site protease 1 and 2 (sp1, sp2) and the cleaved form activates the expression of further chaperones and other downstream genes [22,23]. Both IRE1and ATF6 activation result in the up-regulation of GRP78 and as such increased levels of GRP78 are a hallmark of UPR induction [22,26-30].

Although the UPR is primarily a cell survival mechanism [22], where the stress is unrelieved, apoptosis, mediated by cross talk between the ER and mitochondria, is activated [31,32]. At least four pathways have been documented which act respectively through caspase 12 [33,34], CHOP (CCAAT/Enhancer-Binding Protein Homologous Protein) [35,36], JNK (C-Jun N-terminal kinase) [37] and Ca^2+^[38,39]. In addition to the UPR response, the Noxa/PUMA pathway which is transcriptionally regulated by p53 is also activated under conditions of ER stress and leads to the eventual induction of apoptosis [40].

This study sought to comprehensively characterize the induction of apoptosis in DENV 2 infected HepG2 cells, and to determine whether there was activation of multiple ER stress pathways consistent with our previous observations in monocytic cells [19].

## Results

### Activation of ER stress response pathways

**Figure 1:**
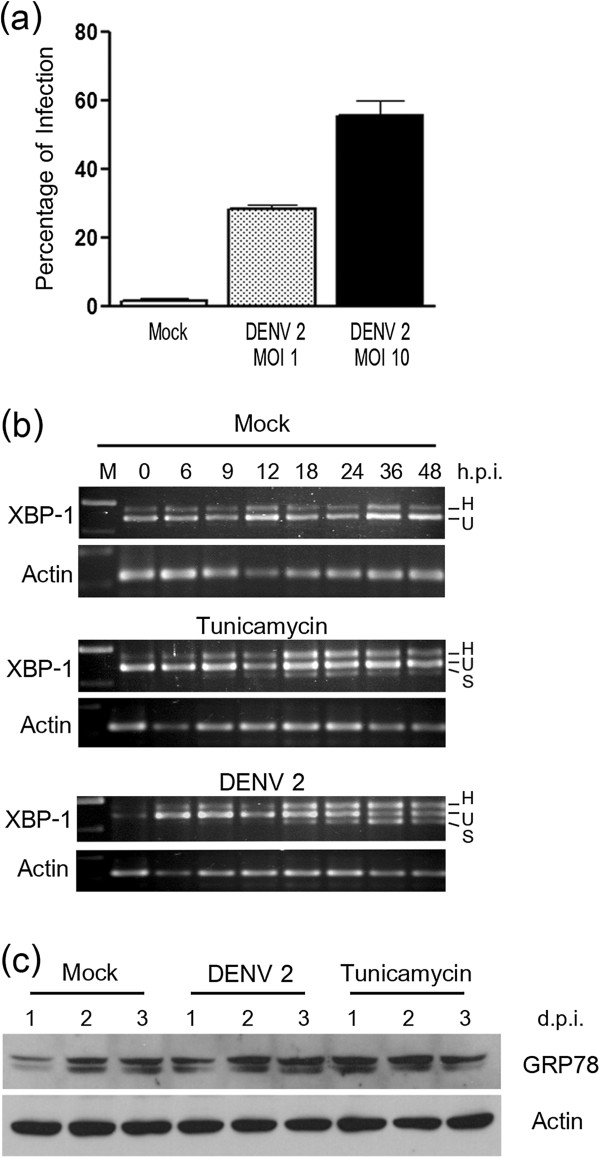
**ER stress in DENV 2 infected HepG2 cells. (a)** HepG2 cells were either infected with DENV 2 at m.o.i. 1 or 10 or mock infected and examined for the percentage of infected cells at 24 hours post infection by flow cytometry. **(b)** and **(c)** HepG2 cells were either infected at m.o.i. 10 or mock infected or treated with tunicamycin and after the times indicated examined for the expression of **(b)** XBP-1 and actin by RT-PCR or **(c)** GRP78 by western blotting.

In our previous study we observed that experiments on activation of the UPR are prone to misinterpretation when undertaken on cell cultures with a low percentage of infection. We therefore initially determined the percentage of infected HepG2 cells after infection with DENV-2 at m.o.i.s of 1 and 10. At 24 hours post infection, percentage infection was determined by flow cytometry after staining with an antibody directed against DENV E protein. Results (Figure 1a) showed that approximately 30% of cells were infected after infection at m.o.i. 1, while nearly 60% of cells were infected when m.o.i 10 was used. An m.o.i. of 10 was used in all subsequent experiments.

To investigate the activation of the UPR pathway in response to DENV infection, DENV 2 (strain 16681) was used to infect HepG2 cells under standard conditions at 10 p.f.u./cell in parallel with mock infected and tunicamycin treated cells. Cells were harvested at various time points and examined for the presence of the ER stress specific splicing product of XBP-1 by RT-PCR. Results show that both tunicamycin treated and DENV 2 infected HepG2 cells showed the presence of the spliced product of XBP-1 (Figure 1b) while no spliced product was seen in mock infected cells. The presence of a heteroduplex product was seen in all cells (tunicamycin treated, mock and dengue infected) as has been noted by others [41-43]. The presence of the spliced product of the XBP-1 transcript demonstrate the activation of the UPR by DENV 2 infection, in agreement with the previous study of Umareddy and colleagues [43] in A549 (human alveolar basal epithelial) cells, and Klomporn and colleagues in U937 monocytic cells [19].

As up-regulation of GRP78 is a common hallmark of UPR activation induction, [22,26-30] and in particular is up-regulated by the activation of XBP-1 [44], the level of GRP78 was examined by western blot analysis. Results showed the over-expression of GRP78 in response to DENV 2 infection (Figure 1c) as has been noted by others [45].

**Figure 2:**
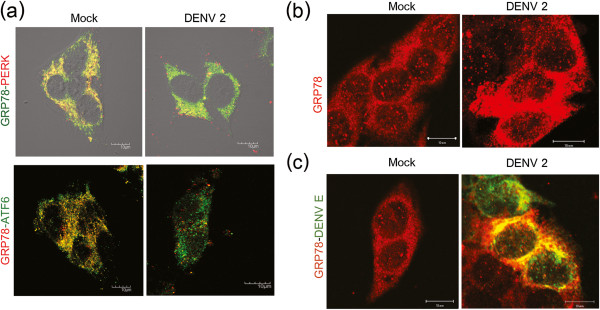
**Activation of the UPR in DENV 2 infected HepG2 cells.** HepG2 cells were either infected with DENV 2 at m.o.i. 10 or mock infected and examined at 24 hours post infection by confocal microscopy for the expression of **(a)** GRP78 and PERK or GRP78 and ATF6 or **(b)** GRP78 alone or **(c)** GRP78 and DENV E protein. For **(a)** and **(c)** representative merged images are shown.

We next examined whether the UPR sensor molecule PERK was found in association with GRP78 (Figure 2a). As would be expected, in mock infected cells a high degree of colocalization was observed between GRP78 and PERK (mean Pearson correlation coefficient 0.75, 95% CI 0.71-0.79). Infection with DENV 2 significantly decreased the degree of colocalization (mean Pearson correlation coefficient 0.50, 95% CI 0.47-0.55; P < 0.001). Similarly, a significantly lower colocalization was observed between GRP78 and ATF6 in DENV 2 infected cells (mean Pearson correlation coefficient 0.42, 95% CI 0.40-0.46 p < 0.001) in comparison to mock infected cells (mean Pearson correlation coefficient 0.8, 95% CI 0.78-0.84) (Figure 2a).

Confocal analysis supported the western blot analysis showing over-expression of GRP 78 (Figure 2b). As previous reports have shown an interaction between GRP78 and DENV 2 E protein [46-48] and we have proposed that this interaction is serotype specific [46,48] we therefore investigated the colocalization of DENV 2 E protein in relationship to GRP78. A high level of colocalization between GRP78 and DENV 2 E protein was observed in DENV 2 infected samples (mean Pearson correlation coefficient 0.70) (Figure 2c).

**Figure 3:**
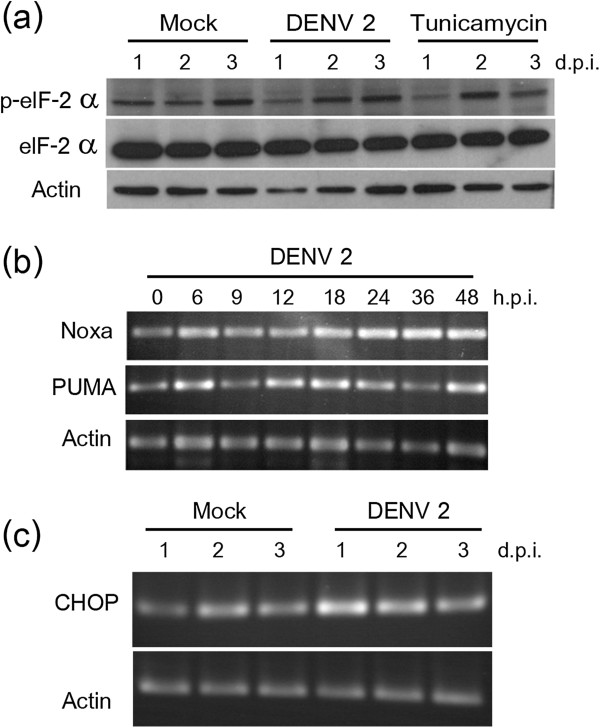
**Activation of ER stress responses in DENV 2 infected HepG2 cells.** HepG2 cells were either infected at m.o.i. 10 or mock infected or treated with tunicamycin and after the times indicated examined for the expression of **(a)** p-eIF-2α, eIF-2α and actin by western blotting or **(b)** Noxa, PUMA and **(c)** CHOP by RT-PCR.

Dissociation of PERK from GRP78 leads to oligomerization and activation of the cytosolic kinase domain which leads to the subsequent phosphorylation of eIF-2α. The phosphorylation of eIF-2α was therefore investigated in DENV 2 infected HepG2 cells in parallel with tunicamycin treated control cells. Results (Figure 3a) showed an initial downregulation of phosphorylation of eIF-2α and an increase on days 2 and 3 p.i. Similarly an initial down regulation of phosphorylation of eIF-2α was observed in tunicamycin treated cells, but higher levels were observed on day 2 post treatment.

### Noxa, PUMA and CHOP

**Figure 4:**
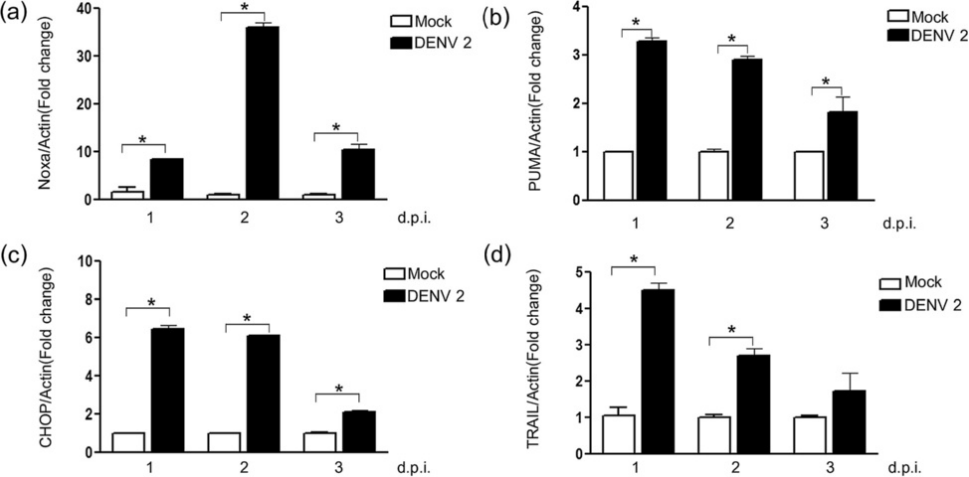
**Quantitative real time PCR analysis of expression of Noxa, PUMA, CHOP and TRAIL.** HepG2 cells were either infected at m.o.i. 10 or mock infected and after the times indicated examined for the expression of **(a)** Noxa, **(b)** PUMA, **(c)** CHOP and **(d)** TRAIL by quantitative real time PCR. The relative expression levels of CHOP, Noxa, PUMA and TRAIL were normalized against actin using the comparative CT method (2^-∆∆CT^ method). * indicates p < 0.05.

Noxa and PUMA are transcriptionally regulated ER stress response genes that promote the induction of apoptosis. Expression of these two genes was examined by semi-quantitative RT-PCR. A clear induction of both genes was seen (Figure 3b), although induction of PUMA appeared to occur somewhat before the induction of Noxa. Similarly, a clear induction of CHOP was observed as early as 24 hours post infection (Figure 3c). These results were confirmed by real time PCR and expression of all three genes was shown to be significantly up regulated (See Figure 4). The delayed expression of Noxa as compared to PUMA was confirmed by the real time PCR analysis.

### Apoptosis

**Figure 5:**
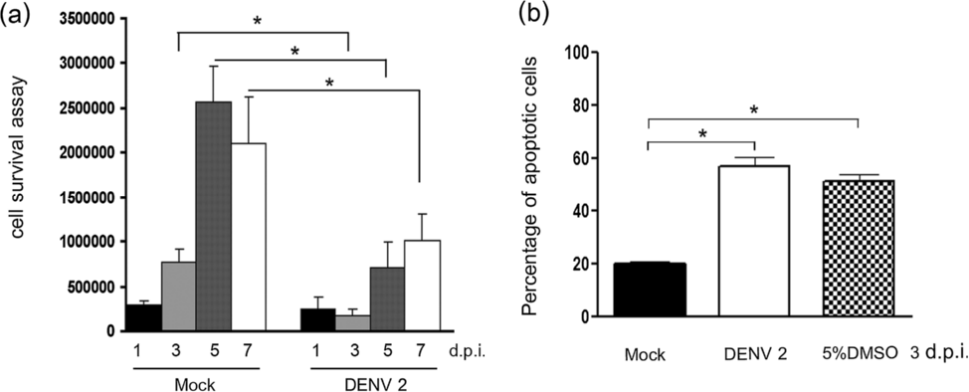
**Cell viability in DENV 2 infected HepG2 cells.** HepG2 cells were mock infected or infected with DENV 2 at m.o.i 10 and analyzed for **(a)** cell viability by trypan blue exclusion assay on days 1, 3, 5 and 7 p.i., **(b)** the percentage of apoptotic cells as assessed by AnnexinV/propidium iodide double staining and analysis by flow cytometery on day 3 p.i.

**Figure 6:**
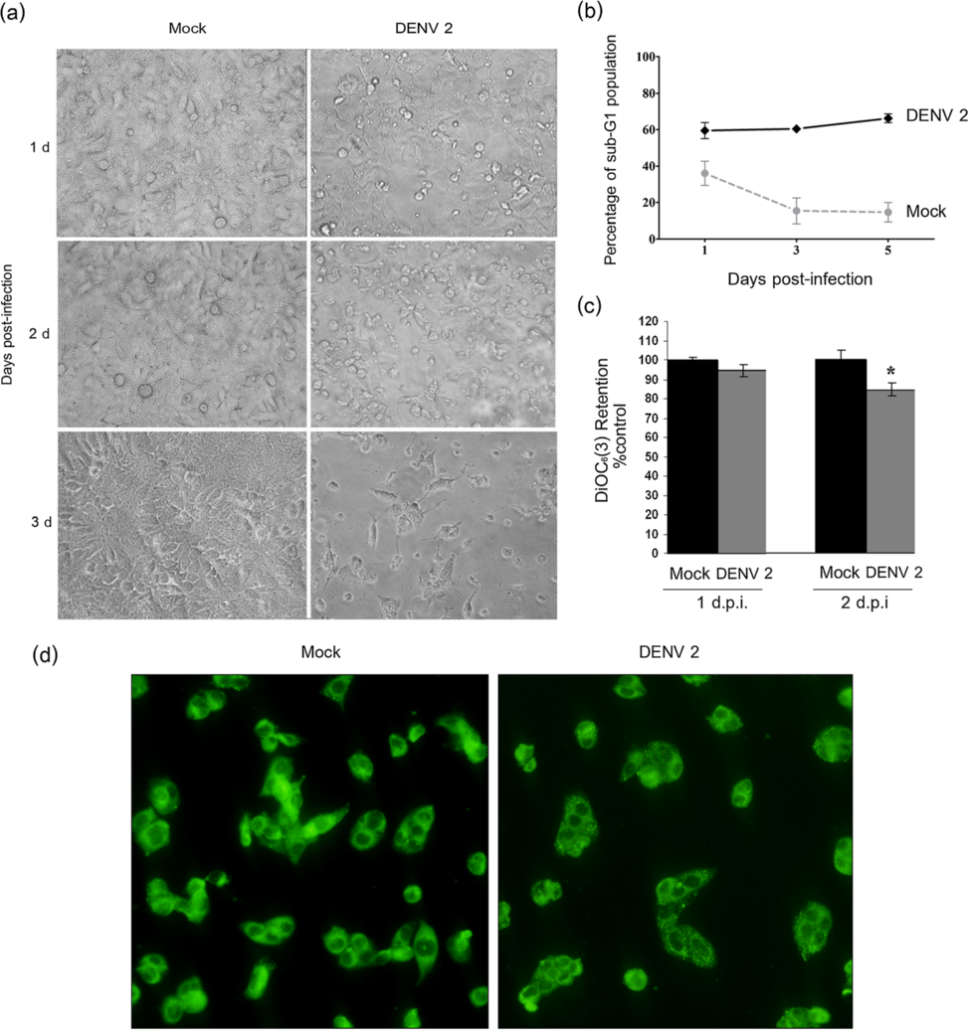
**Apoptosis in DENV 2 infected HepG2 cells.** HepG2 cells were mock infected or infected with DENV 2 at m.o.i 10 and analyzed for **(a)** cell morphology by light microscopy on days 1 to 3 p.i., **(b)** the presence of a sub-G1 DNA population as assessed by flow cytometry after staining with propidium iodide for days 1 to 5 p.i., **(c)** membrane mitochondrial potential after staining with DiOC_6_(3) and analysis by fluorescent spectrophotometery on days 1 and 2 p.i. with additional direct observation of the cells under a fluorescent microscope **(d)**. *indicates p < 0.05.

In a previous study [18] we documented the induction of apoptosis in DENV 2 infected HepG2 cells through the observation of chromatin condensation, the presence of a DNA ladder and increased Annexin V/propidium iodide staining as well as a reduction in cell numbers in infected cultures as opposed to mock cultures. As that study was undertaken at a lower multiplicity of infection than the present study, we initially undertook a cell viability assay to determine whether a deficit in cell number was seen under the higher infection conditions, and whether this was associated with increased numbers of apoptotic cells as assessed by Annexin V/propidium iodide staining. Results (Figure 5) confirmed our previous observations at the lower multiplicity of infection. We further documented clear morphological changes in DENV 2 infected HepG2 cells as opposed to mock infected cell (Figure 6a).

To confirm DNA fragmentation, cells were directly analyzed for DNA content by flow cytometry after propidium iodide staining and analyzed for the presence of a sub-G1 population. Results (Figure 6b) show that a significantly increased sub-G1 population was present in infected cells as early as 24 hours post infection.

### Mitochondrial transmembrane potential (Δψ_m_) in DENV 2 infection

Loss of mitochondrial function is a common hallmark of apoptosis and can be observed by a decrease in mitochondrial membrane potential. To determine whether there was a loss of mitochondrial membrane potential in response to DENV infection, HepG2 cells were either mock infected, or infected with DENV 2 at 10 p.f.u./cell. At 24 or 48 hours post infection cells were incubated with DiOC_6_(3) for 30 minutes. The cells were harvested and washed twice with PBS prior to being lysed and homogenized in deionized water. The concentration DiOC_6_(3) retained was measured by fluorescent spectrophotometery at 488 nm excitation and 500 nm emission. The retention of DiOC_6_(3) was compared against mock-infected cells as control. DENV 2 infected cells showed a significant (p < 0.05) reduction in mitochondrial transmembrane potential (Δψ) at 48 hours post infection (Figure 6c). Direct microscopic examination of DiOC_6_(3) stained cells showed an increased punctuate staining pattern as opposed to mock infected cells (Figure 6d).

### Activation of caspases in DENV 2 infected HepG2 cells

**Figure 7:**
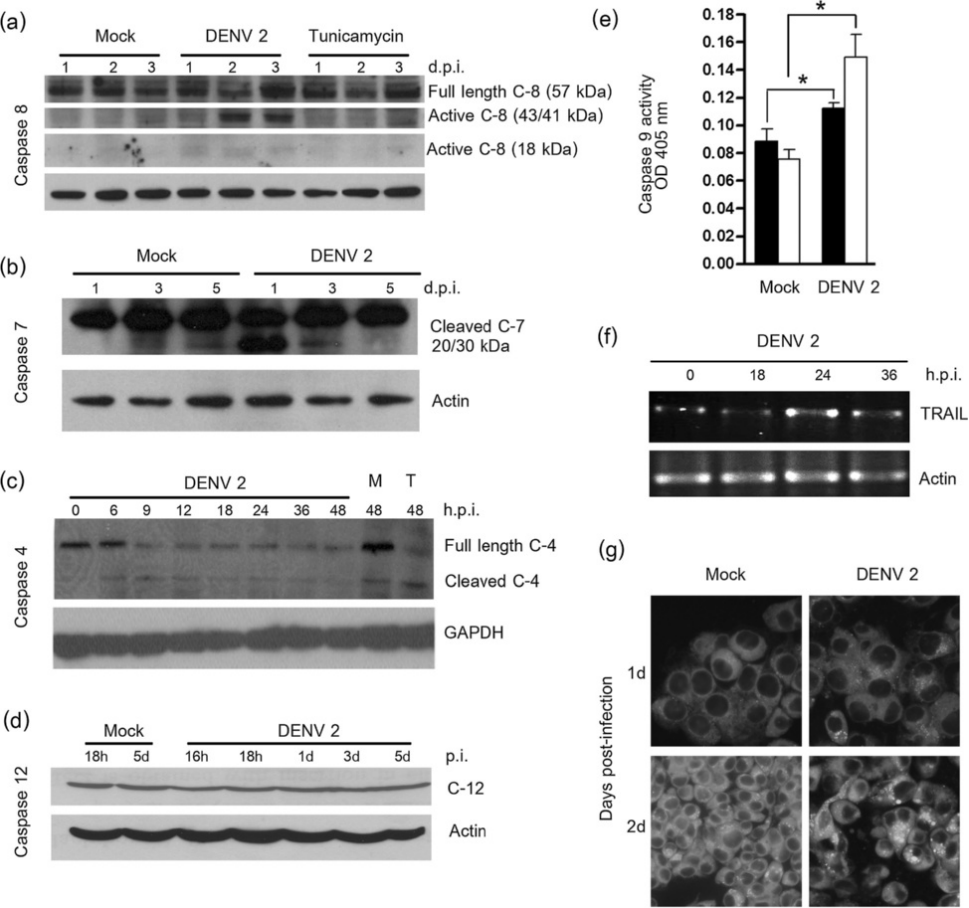
**Pathways of apoptosis induction in DENV 2 infected HepG2 cells.** HepG2 cells were either infected at 10 p.f.u./cell or mock infected and examined at the times indicated by **(a, b, c, d)** Western blot for the activation of caspases 4, 7, 8 and 12 or **(e)** by ELISA for activation of caspase 9 (solid bars day 1 p.i., open bars day 2 p.i.). **(f)** DENV infected HepG2 cells were examined for the expression of TRAIL by RT-PCR and **(g)** by microscopy after staining with MDC for the presence of autophagic vacuoles. * indicates p < 0.05.

To investigate the proteolytic activation of caspases, mock-infected or DENV 2 infected cells were harvested at various time points post-infection and total protein was extracted and then separated by electrophoresis on 15% SDS-polyacrylamide gels. The proteins were subsequently either transferred to nitrocellulose membranes and probed with antibodies against caspases 4, 7, 8 and 12 or used in an ELISA assay to determine the activation of caspase 9. For the western analysis, each antibody is able to detect both procaspase and active cleaved forms of the caspases and membranes were subsequently probed with a mouse monoclonal anti-actin or anti GAPDH antibody as an internal control. Western analysis (Figure 7a-d) showed the proteolytic cleavage of caspases 4, 7 and 8 in DENV 2 infected cells indicated by the presence of active forms of these proteins, while the ELISA assay confirmed the activation of caspase 9 seen in response to DENV 2 infection of HepG2 cells (p < 0.05; Figure 7e). As activation of all examined caspases was seen in DENV 2 infected cells, we also examined the expression of caspase 12 as a control. In human cells and cell lines, caspase 12 is normally inactive due to the inheritance of mutated alleles of this protein [49]. As expected, no activation of caspase 12 was seen in response to DENV 2 infection (Figure 7d) suggesting that activation of caspases 4, 7, 8 and 9 was a direct and specific consequence of DENV 2 infection.

### Expression of TRAIL

We have previously documented increased expression of TRAIL in both HepG2 cells and in primary hepatocytes in response to DENV 2 infection [13]. Because of the recent retraction of the seminal paper postulating the activation of TRAIL as a critical component of the extrinsic activation of apoptosis in response to DENV infection of liver cells [50], we reconfirmed the increased expression of TRAIL in response to DENV 2 infection. Results (Figure 7f) showed an increase in TRAIL expression in response to DENV 2 infection, consistent with our earlier report [13]. The significant an early increase in TRAIL expression was confirmed by real time PCR (see Figure 4).

### Induction of autophagy in response to DENV 2 infection

We have previously extensively documented the induction of autophagy in response to DENV 2 infection of HepG2 [51] and other cells [52]. To confirm the activation of autophagy in response to DENV 2 in this study, HepG2 cells were either mock infected or infected with DENV 2 and stained with the acidotropic dye monodansylcadaverine (MDC) and examined under a fluorescent microscope on days 1 and 2 p.i. Results showed a notable increase in MDC positive vacuoles (Figure 7g), consistent with the activation of autophagy in DENV 2 infected HepG2 cells as documented previously [51].

## Discussion

The involvement of the liver in dengue infections has been the subject of some controversy. However, a significant amount of evidence from primary cells, animal model studies and *in vitro* experiments suggests that the liver is directly involved in the pathogenesis of the disease, and that hepatocytes are a *bona fide* target of DENV (reviewed in [53]). Similarly, several studies in both primary [13] and transformed [16-18,54-58] liver cells have documented the induction of apoptosis in response to infection with DENV. However, the induction of apoptosis has been proposed to occur by different groups by both intrinsic (mitochondrially mediated) and extrinsic (death receptor mediated) pathways. The results seen here, specifically the activation of both caspases 8 and 9, the decrease of mitochondrial membrane potential and the up regulation of TRAIL support a model in which both intrinsic and extrinsic pathways are activated, similar to our recent report on monocytic cells where both intrinsic and extrinsic apoptosis pathways were independently activated [19]. As such, the activation of multiple, independent apoptosis pathways in response to DENV infection maybe a common mechanism, irrespective of cell type. The activation of multiple, independent apoptosis pathways in liver cells in response to DENV infection would also tend to unify the disparate studies that propose activation only through one specific pathway via one of several proposed mechanisms [16,17,56-58].

In a study in 2008 Nasirudeen and Liu [16] proposed that apoptosis in liver cells occurred via the p53 dependent activation of mitochondrially mediated (i.e. intrinsic) apoptosis. However, the proposal of p53 playing a significant role in the induction of apoptosis in liver cells is inconsistent with an earlier study which had shown the robust induction of apoptosis in the p53 negative cell line Hep3B [18].

However, as shown here, both Noxa and PUMA, which are ER stress response genes transcriptionally regulated by p53, are up-regulated in response to infection and therefore where p53 is functional in a cell, it may well play a role in mediating the apoptotic response. However, even in the absence of p53, apoptosis can still occur through the activation of non-p53 dependent pathways, again supporting our previous contention in monocytic cells that apoptosis is induced by multiple independent pathways [19].

More recently Nasirudeen and Liu proposed that caspase 1 is critical to the induction of apoptosis [17] in DENV infected cells. Caspase 1 is a human “inflammatory caspase” together with caspases 4, 5 and 12, although caspase 12 is normally inactive in humans [49]. Caspase 1 is activated by association with the so called “inflammasomes” which are large oligomeric complexes that assemble in response to signals such as the sensing of pathogen associated molecular patterns (PAMPS) or the presence of danger associated molecular patterns [59]. Recent evidence has suggested that caspase 1 activation requires the proceeding activation of caspase 4 [60] and earlier studies have suggested that caspase 4 is localized to the ER membrane and that it may be activated directly by ER stress [61]. In this way, the activation of caspase 4 as a result of ER stress and the subsequent activation of caspase 1 (with or without association of the inflammasome) might represent yet another independent pathway by which DENV infection results in apoptosis.

In other studies, the dengue capsid protein has been implicated as playing a role in the mediating the induction of intrinsic apoptosis in liver cells [55,56,58]. Limjindaporn and colleagues proposed that the nuclear interaction between the DENV capsid protein and the death domain associated protein Daxx is essential for the induction of apoptosis [56,58], and subsequently that the DENV capsid protein induces apoptosis through the action of either CD137 or RIPK2 [57]. However, a recent study by Jianling and colleagues [55] proposed that the interaction between DENV capsid protein and the calcium modulating cyclophilin-binding ligand (CAML) serves to subvert the apoptotic process. The contradictory results suggesting that the DENV capsid protein either promotes [56,58] or inhibits [55] apoptosis suggests that further research is required to define the function of this protein in the apoptotic process.

The induction of extrinsic apoptosis in DENV infected liver cells was originally proposed in a study that has now been retracted [50]. In the original study it was proposed that DENV infection resulted in the up-regulation of TRAIL which interacted with the Apo2L/TRAIL receptor DR5/TRAIL-R2 expressed on the surface of liver cells. This model was subsequently supported by our studies in both primary and transformed cells which showed the increased expression of TRAIL [13] as re-confirmed here. Elevated levels of TRAIL in response to DENV infection have been documented for human primary monocytes, B cells and dendritic cells [62] as well as for HUVECs (human umbilical vein endothelial cells) [63] and elevated levels of TRAIL were found in DENV infected patients in the febrile phase as compared to normal controls [62]. Warke and colleagues observed that TRAIL expression reduced viral titers in DENV infected HUVECs, and they proposed that TRAIL played an antiviral role in an apoptosis independent manner [63]. However, as was observed in the now retracted Matsuda study [50], liver cells express the Apo2L/TRAIL receptor DR5/TRAIL-R2 and upon binding of TRAIL the clustered DR5 receptor complex recruits, through its cytoplasmic domain the adapter molecule FADD (Fas-associated death domain) which in turn recruits pro-caspase 8, forming a death induced signaling complex (DISC) which directly results in the activation of caspase 8 [64,65]. Our results showing the increased expression of TRAIL and the activation of caspase 8 would therefore support the extrinsic induction of apoptosis in liver cells mediated by TRAIL, and suggests that the interaction between TRAIL and DENV infected cells is cell type specific. In this way, the elevated levels of TRAIL seen in serum of febrile DENV infected patients [62] could serve to both remove infected liver cells and protect other cells such as endothelial cells through its antiviral activities [63].

Interestingly, we observed a significant activation of caspase 9 on day 1 post infection, while caspase 8 was shown to be activated from day 2 post infection. Caspase 7 which is activated by both intrinsic and extrinsic pathways showed significant levels of activation from day 1, and less thereafter. The delayed activation of caspase 8 (as compared to caspase 9) would also support activation of this pathway through TRAIL, which showed significant levels of expression only after 24 hours infection. Caspase activation was predominantly shown to be an early event, with apparently less activation at the later stages. However, as the cells are constantly dying as a consequence of the infection, the actively infected cells may represent a smaller and smaller proportion of the cells in culture as the non-infected cells may outgrow the infected cells.

Induction of the UPR as a consequence of DENV infection in several cell lines has been well documented [19,43,66,67] and it is currently thought that it is the influx of nascent unfolded proteins to the ER as a consequence of infection is the critical event in triggering the UPR [67] and, as shown here for the first time, the UPR is activated in response to DENV infection of liver cells. Prolonged activation of the UPR is known to trigger apoptosis through a number of pathways [33,34,36-39], several of which lead directly to mitochondrially mediated (intrinsic) apoptosis and, as documented here by cleavage of caspase 9 and changes in mitochondrial membrane potential, mitochondrially mediated apoptosis is induced in DENV infected liver cells. The observation of reduced mitochondrial membrane potential in DENV infected liver cells is consistent with the observations of others [54].

Activation of PERK leads to the phosphorylation of the translation initiation factor eIF-2α, which leads to the attenuation of translation initiation with the exception of ATF4 and its downstream target CHOP (also known as GADD153) whose expression is increased when eIF-2α is phosphorylated [22,68], all of which were observed to occur under conditions of DENV infection of HepG2 cells. Critically, prolonged expression of CHOP, which is also a target of the other branches of the UPR [22,68] results in the induction of apoptosis through a number of potential pathways, such as through the Bcl2 family members or through the ERO1α–IP3R–Ca2 + −CaMKII pathway both of which end in mitochondrially mediated apoptosis [69]. In a recent study Pena and Harris [70] investigated the induction of the unfolded protein response in response to DENV infection in human fibrosarcoma 2fTGH cells and in a number of knockout mice embryonic fibroblast (MEF) cell lines. Pena and Harris reported the time dependent modulation of the UPR, but predominantly focused on the first 12 hours of infection [70]. In particular they reported a peak of phosphorylation of eIF2α at 6 hours post infection after which levels returned to mock levels, while in this study we see a down regulation at 24 hours, and a significant increase on days 2 and 3 p.i. Somewhat surprisingly, while Pena and Harris report an increase in expression of CHOP, they report no activation of caspase 9 or and indeed, no induction of apoptosis in response to infection [70]. Given that apoptosis in response to dengue infection has been reported in numerous studies in a number of different cell types [9,17-19,57,71-80] the significance of their observations remains unclear.

Studies have shown that activation of the UPR can lead to the induction of apoptosis through activation of caspase 12 [33,34]. However, caspase 12 in humans is predominantly inactive due the high occurrence of inactivating mutations in this gene in the human population [49]. As shown here, no activation of caspase 12 was seen in response to DENV infection consistent with its inactive status, however the lack of processing seen for caspase 12 confirms the specificity of activation of the other caspases examined in this study.

Induced ER stress and the activation of the UPR has been well characterized as an inducer of autophagy [81-83]. We and others have previously extensively characterized the induction of autophagy in liver cells in response to DENV infection [51,84-86], and in this study showed increased staining with MDC in DENV infected cells. MDC is a fluorescent acidotropic dye that was originally believed to specifically label autophagic vacuoles [87]. Subsequent investigations have suggested that MDC stains vacuoles late in the autophagic process [88]. While not a rigorous analysis of autophagy [89], it serves to confirm our earlier studies in the same cell line and with the same virus [51,85]. Autophagy is believed to be induced in response to ER stress as an attempt to decrease the stress through increased degradation of misfolded proteins [90], and studies have suggested that autophagy can be induced through either PERK [90,91] or IRE1 [90,92]. By relieving ER stress, autophagy can therefore act in a pro-survival manner and can inhibit the onset of apoptosis [93]. However, studies have shown that prolonged activation of autophagy can promote cell death through both apparently apoptosis dependent and apoptosis independent mechanisms [93].

## Conclusions

Our results show that in response to DENV infection of liver cells, there is the activation of multiple ER stress pathways and multiple modes of cell death, namely the activation of intrinsic, extrinsic and possibly inflammasome mediated apoptosis pathways, as well as the activation of autophagy which can further lead to (intrinsic) apoptosis dependent and apoptosis independent cell death. These results suggest that removal of DENV infected cells from the liver is ensured through the activation of multiple pathways.

## Methods

### Viruses, cells and treatments

The human hepatoma cell line HepG2 (ATCC No. HB-8065) was cultivated as described previously [94]. Dengue virus serotype 2 (DENV 2; strain 16681) was propagated in the *Aedes albopictus* derived cell line C6/36 (ATCC No. CRL-1660). The virus was partially purified by centrifugation to remove cell debris and stored frozen at −80°C. Virus titer was determined by standard plaque assay as described elsewhere [95]. HepG2 cells were treated with 2 μg/ml tunicamycin (T7765, Sigma-Aldrich, Milwaukee, WI) as indicated.

### Cell morphology and viability

HepG2 cells were seeded for 24 hours under standard growth condition, cells were either mock-infected or infected at 10 p.f.u./cell with DENV 2 for up to 7 days post infection. On days 1 to 3 p.i., cells were examined directly under an inverting light microscope (Nikon Eclipse TS100, Nikon Instruments Inc., Melville, NY) and on days 1, 3, 5 and 7 p.i. the live cell number was determined by a trypan blue exclusion assay. The experiment was undertaken independently in triplicate for each day.

### Semi-quantitative RT-PCR

Total RNA was isolated using TRI reagent (Molecular Research Center, Inc., Cincinnati, OH) according to the manufacturer’s instructions. RNA was transcribed to cDNA using ImProm-II™ reverse transcriptase (Promega, Madison, WI). cDNA amplifications for XBP-1, Noxa, PUMA, TRAIL, CHOP and actin were undertaken exactly as previously described, using the primers and cycle conditions described previously [13,19]. All products were analyzed on 2% agarose gels.

### Quantitative real time PCR

Mock infected or HepG2 cells infected with DENV 2 at m.o.i. 10 were collected at the indicated time points and total RNA extracted using TRI Reagent (Molecular Research Center, Inc., Cincinnati, OH). Dnase I (Promaga, Madison, WI) was use to remove genomic DNA. Subsequently 1 μg/mL of the RNA was use to obtain cDNA using Oligo (dT) (Bio Basic, Inc., Ontario, Canada) and Improm-II ™ reverse transcriptase enzyme (Promega, Madison, WI). Quantitative real time PCR reactions were performed based on SYBR technique by using the KAPA SYBR FAST qPCR Kit 2X Master MIX (Kapa Biosystems Inc, Woburn, MA) in a Mastercycler ep realplex real time PCR system. Reactions were undertaken with an initial 3 minutes at 95°C, followed by denaturation at 95°C for 10 secs, annealing at 60°C for 30 secs and extention at 72°C for 20 secs for 40 cycle. Primers used were CHOP (CHOPfw: 5′-ACCAGGAAACGGAAACAGAGTGGT-3′) and (CHOPrv: 5′-TCCTGCTTGAGCCGTTCATTCTCT-3′) Noxa (Noxafw: 5′-AGTCGAGTGTGCTACTCAACTCAG-3′) and (Noxarv: 5′-AGGTTCCTGAGCAGAAGAGTTTGG-3′) PUMA (PUMAfw: 5′-ACGACCTCAACGCACAGTACGA-3′) and (PUMArv: 5′-TAATTGGGCTCCATCTCGGG-3′) TRAIL (TRAILfw: 5′- CAACTCCGTCAGCTCGTTAG-3′) and (TRAILrv: 5′- TGCCCACTCCTTGATGATTC -3′) and Actin (Actinfw :5′-ACCAACTGGGACGACATGGAGAAA-3′) and (Actinrv: 5′-TAGCACAGCCTGGATAGCAACGTA-3′). The relative expression levels of CHOP, Noxa, PUMA and TRAIL were normalized against actin using the comparative CT method (2^-∆∆CT^ method).

### Fluorescence confocal microscope imaging and quantitation

Cells grown on cover slips were washed twice with 1x PBS followed by immersion in 100% ice-cold methanol for 20 minutes. Cells were subsequently washed twice with 1xPBS before incubation for 10 minutes with 1xPBS containing 0.3% Triton-X100. Cells then were blocked with 5% FBS in 1xPBS containing 0.03% Triton-X100 for 1 hour at room temperature. Cells were subsequently incubated overnight with two appropriate primary antibodies at 4°C followed by incubation with two appropriate secondary for 1 hr at room temperature followed by three washes with 1x PBS containing 0.03% Triton-X100 before mounting. Antibodies used were a 1:10 dilution of rabbit polyclonal anti GRP78 antibody (sc-13968; Santa Cruz Biotechnology Inc., Santa Cruz, CA.) followed by either a 1:50 dilution of a Rhodamine Red X conjugated goat anti rabbit IgG antibody (111-295-144; Jackson, West Grove, PA) or a 1:300 dilution of a FITC conjugated donkey anti rabbit IgG antibody (sc-2090; Santa Cruz Biotechnology Inc.), a 1:10 dilution of a goat polyclonal anti GRP78 antibody (sc-1050; Santa Cruz Biotechnology, Inc) followed by a 1:100 dilution of a Cy5 conjugated rabbit anti goat IgG antibody (81–1616; Invitrogen, Grand Island NY), a 1:200 dilution of mouse monoclonal anti dengue complex (MAB8705, Chemicon, EMD Millipore Corporation, Billerica, MA CA) followed by a 1:10 dilution of a FITC conjugated goat anti mouse IgG antibody (02-18-06; KPL, Guilford, UK), a 1:50 dilution of a mouse monoclonal anti ATF6 antibody (IMG-273; Imgenex, San Diego, CA) followed by a 1:10 dilution of a FITC conjugated goat anti mouse IgG (02-18-06; KPL, Gaithersburg, MD), a 1:50 dilution of a goat polyclonal anti PERK antibody (sc-9481; Santa Cruz Biotechnology, Inc.) followed by a 1:100 dilution of a Cy5 conjugated rabbit anti-goat IgG (81–1616; Invitrogen).

Fluorescently labeled cells were observed under an Olympus FluoView 1000 (Olympus Corporation, Shinjuku-ku, Tokyo) confocal microscope equipped with Olympus FluoView software v. 1.6 or a Carl Zeiss Laser scanning system LSM510 (Carl Zeiss Advanced Imaging Microscopy, Jena, Germany) equipped with Zeiss LSM5 Image Browser software version 3.2.0115. Images were recorded in 3 channels. Fifteen fields were examined for each experiment and representative results shown. Pearson correlation coefficients for co-localization were determined as described elsewhere [51].

### Fluorescent microscopy

For monodansylcadaverine (MDC) and 3,3′-dihexyloxacarbocyanine Iodide (DiOC_6_(3)) staining, HepG2 cells infected with DENV 2 at 10 pfu/cell or mock infected were incubated with 0.05 mM MDC in PBS at 37°C for 1 hour or 100 nM DiOC_6_(3) (Sigma-Aldrich, Milwaukee, WI) for 30 minutes before examination under a fluorescence microscope (Olympus BX61, Olympus).

### Sub G1 analysis

To determine the DNA fragmentation upon infection in DENV 2 infected cells, HepG2 cells were either mock infected or infected with DENV 2 at 10 p.f.u./cell and incubated for up to 5 days p.i. Cells were collected on the indicated days post infection and washed with 1x PBS. Cells were then fixed with 70% ethanol in 1xPBS. After being washed with 1xPBS, cells were treated with 10 mg/ml RNase A for one hour, and then incubated with 1 mg/ml propidium iodide (PI) for 15 minutes before analysis by flow cytometry (BD FACSCalibur, BD Biosciences, San Jose, CA). Experiments were undertaken independently in triplicate. In data analysis cell debris was gated out and the R1 population plotted in histogram form.

### Western blot analysis

Mock infected or dengue infected cells were collected by scraping at various times post infection and total proteins extracted and subjected to western blot analysis exactly as described previously [19,51,85]. Membranes were blocked with 5% skim milk in TBS-T at room temperature for 1 hr and subsequently incubated for 2 hr with an appropriate primary antibody, followed by incubation with an appropriate secondary antibody for 1 hr at room temperature.

Primary antibodies used were mouse monoclonal antibodies against caspase 8 (1C12, Cell Signaling Technology, Inc., Danvers, MA), caspase 7 (C7, Cell Signaling Technology), actin (sc-8432, Santa Cruz Biotechnology Inc.), glyceraldehyde 3-phosphate dehydrogenase (GAPDH) (sc-32233, Santa Cruz Biotechnology, Inc.) and dengue nonstructural protein 1 (NS1) (ab41616, Abcam plc, Cambridge, UK) followed by a horseradish peroxidase (HRP)- conjugated rabbit anti-mouse IgG (A9044, Sigma-Aldrich) as well as rabbit polyclonal antibodies against GRP78 (SC-13968, Santa Cruz Biotechnology Inc.), phospho-eIF-2α (9721, Cell Signaling Technology), eIF-2α (9722, Cell Signaling Technology), caspase 12 (C2088-58, United States Biological, Swampscott, MA) followed by a horseradish peroxidase conjugated goat anti-rabbit IgG (31460, Pierce, Rockford, IL) as well as goat polyclonal antibodies directed against caspase 4 (ab27485, Abcam plc) and actin (sc-1616, Santa Cruz Biotechnology Inc.) followed by a horseradish peroxidase conjugated donkey anti-goat IgG (PA1-86326, Pierce).

The signals were developed using the ECL Plus Western Blotting Analysis kit (Amersham Pharmacia Biotech, Piscataway, NJ).

### Mitochondrial transmembrane potential (Δψ_m_) measurement

HepG2 cells either mock infected or infected with DENV 2 at 10 p.f.u./cell were incubated with 100 nM 3,3′-dihexyloxacarbocyanine iodide (DiOC_6_(3)); Sigma-Aldrich, Milwaukee, WI) for 30 min after which cells were washed with PBS and then harvested by centrifugation at 700 × *g* for 5 min. The supernatant was removed, and the pellet was resuspended and washed again in PBS. The pellet was then lysed by the addition of 600 μl of deionized water followed by sonication. The concentration of retained DiOC_6_(3) was read using a fluorescent spectrophotometer (FP6300, Jasco, Essex, UK) at 488 nm excitation and 500 nm emission.

### Annexin V/propidium iodide double staining

HepG2 cells were either mock-infected or infected with DENV 2 at 10 p.f.u./cell. On day 3 post-infection cells were harvested by centrifugation and washed twice with PBS. The cells were double stained with 5 μl of 20 μg/ml FITC-conjugated annexin V and 10 μl of 50 μg/ml propidium iodide in 400 μl binding buffer (Becton Dickinson, Franklin Lakes, NJ). After 15 min cells were analyzed by flow cytometry (FACSCalibur, BD Biosciences, San Jose, CA) using the CELLQuest™ software (BD Biosciences).

### Caspase 9 assay

A total of 5×10^5^ HepG2 cells were grown in 6-well tissue culture plate for 24 hours prior to mock-infection or infection with DENV 2 at 10 p.f.u./cell. Caspase 9 activity was analyzed using the Caspase 9 Colorimetric Activity Assay Kit (Chemicon, Temecula, CA). Briefly the cells were collected and lysed with kit supplied lysis buffer. Cell lysate was incubated on ice for 10 min prior to centrifuging at 10,000 × *g* for 5 min. Supernatant containing cytosolic extract was transferred to a fresh tube. Protein concentration for each sample was then analyzed by Bradford assay. The sample was incubated with caspase 9 substrate in supplied assay buffer at 37°C for 1 hour. Caspase 9 activity was determined by reading the optical density at 405 nm and normalized according to the protein concentration of each sample.

### Statistical analysis

Data was analyzed using the GraphPad Prism program (GraphPad Software Inc., CA). Statistical analysis of significance was undertaken by Paired sample test using SPSS (SPSS, Inc., Chicago, IL). For all comparisons, a P-value ≤ 0.05 was taken as significant.

## Competing interests

The authors declare they have no competing interests.

## Authors’ contributions

CT, AK and DRS designed the study. CT, AK, SK, NW, CF, PC and PP undertook all experimental work. CT, AK and DRS drafted the initial manuscript and all co-authors reviewed the manuscript for important intellectual content and read and approved the final version.
